# *N*′-(2-Hydroxy­naphthyl­idene)-4-methoxy­benzo­hydrazide

**DOI:** 10.1107/S1600536808008076

**Published:** 2008-03-29

**Authors:** Chun-Bao Tang

**Affiliations:** a Department of Chemistry, Jiaying University, Meizhou 514015, People’s Republic of China

## Abstract

The title Schiff base compound, C_19_H_16_N_2_O_3_, was derived from the condensation reaction of 2-hydr­oxy-1-naphthyl­aldehyde with 4-methoxy­benzohydrazide. The dihedral angle between the benzene ring and the naphthyl ring system is 6.8 (2)°. In the crystal structure, mol­ecules are linked through inter­molecular N—H⋯O inter­molecular hydrogen bonds, forming chains running along the *c* axis.

## Related literature

For related structures, see: Tang (2006 ▶, 2007*a* ▶,*b* ▶,*c* ▶,*d* ▶). For reference structural data, see: Allen *et al.* (1987 ▶).
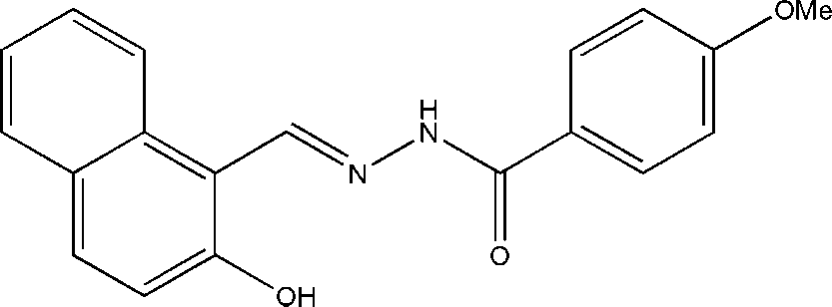


## Experimental

### 

#### Crystal data

C_19_H_16_N_2_O_3_
*M* *_r_* = 320.34
Monoclinic, 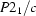

*a* = 11.159 (2) Å
*b* = 15.790 (3) Å
*c* = 8.8300 (18) Å
β = 91.70 (3)°
*V* = 1555.2 (5) Å^3^
*Z* = 4
Mo *K*α radiation
μ = 0.09 mm^−1^
*T* = 298 (2) K
0.32 × 0.32 × 0.30 mm

#### Data collection

Bruker SMART CCD area-detector diffractometer
Absorption correction: multi-scan (*SADABS*; Sheldrick, 1996 ▶) *T*_min_ = 0.971, *T*_max_ = 0.972
13232 measured reflections
3550 independent reflections
2161 reflections with *I* > 2σ(*I*)
*R*_int_ = 0.051

#### Refinement

*R*[*F* ^2^ > 2σ(*F* ^2^)] = 0.056
*wR*(*F* ^2^) = 0.136
*S* = 1.04
3550 reflections
222 parameters
1 restraint
H atoms treated by a mixture of independent and constrained refinement
Δρ_max_ = 0.18 e Å^−3^
Δρ_min_ = −0.20 e Å^−3^

### 

Data collection: *SMART* (Bruker, 2002 ▶); cell refinement: *SAINT* (Bruker, 2002 ▶); data reduction: *SAINT*; program(s) used to solve structure: *SHELXS97* (Sheldrick, 2008 ▶); program(s) used to refine structure: *SHELXL97* (Sheldrick, 2008 ▶); molecular graphics: *SHELXTL* (Sheldrick, 2008 ▶); software used to prepare material for publication: *SHELXL97*.

## Supplementary Material

Crystal structure: contains datablocks global, I. DOI: 10.1107/S1600536808008076/sj2478sup1.cif

Structure factors: contains datablocks I. DOI: 10.1107/S1600536808008076/sj2478Isup2.hkl

Additional supplementary materials:  crystallographic information; 3D view; checkCIF report

## Figures and Tables

**Table 1:** Hydrogen-bond geometry (Å, °)

*D*—H⋯*A*	*D*—H	H⋯*A*	*D*⋯*A*	*D*—H⋯*A*
O1—H1⋯N1	0.82	1.86	2.582 (2)	146
N2—H2⋯O2^i^	0.904 (9)	1.957 (12)	2.834 (2)	163 (2)

Symmetry code: (i) 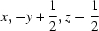.

**Fig. 1.**
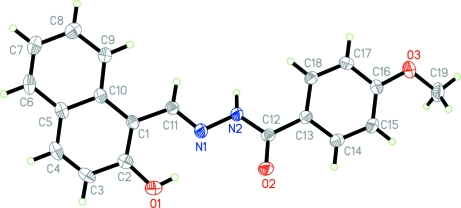
The molecular structure of the compound, showing the atom-numbering scheme. Displacement ellipsoids are drawn at the 30% probability level.

**Fig. 2.**
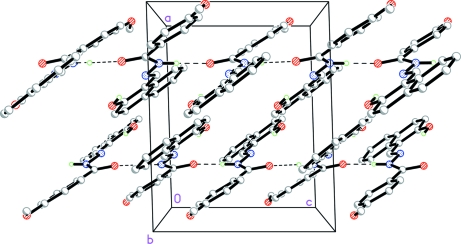
Molecular packing of (I) with hydrogen bonds drawn as dashed lines.

## supplementary crystallographic information

### Comment

Recently, the author has reported the structures of several Schiff base compounds (Tang, 2006, 2007*a*,*b*,*c*,*d*) and, in continuation of work in this area, reports herein the structure of the title compound, (I), Fig. 1, a new Schiff base compound.

In the title compound (Fig. 1), the dihedral angle between the benzene ring and the naphtyl ring is 6.8 (2)°. The torsion angles C1—C11—N1—N2, C11—N1—N2—C12, and N1—N2—C12—C13 are 1.3 (2), 17.0 (2), and 1.5 (2)°, respectively. All the bond lengths are within normal values (Allen *et al.*, 1987).

In the crystal structure of the compound, molecules are linked through N—H···O intermolecular hydrogen bonds (Table 1), forming chains running along the *c* axis (Fig. 2).

### Experimental

2-Hydroxy-1-naphtylaldehyde (0.1 mmol, 17.2 mg) and 4-methoxybenzohydrazide (0.1 mmol, 16.6 mg) were dissolved in an ethanol solution (20 ml). The mixture was stirred at reflux for 10 min to give a clear colorless solution. Colorless needle-like crystals of the compound were formed by slow evaporation of the solvent over several days.

### Refinement

H2 atom was located from a difference Fourier map and refined isotropically, with N—H distance restrained to 0.90 (1) Å. Other H atoms were constrained to ideal geometries, with C—H = 0.93–0.96 Å, O—H = 0.82 Å, and with *U*_iso_(H) = 1.2*U*_eq_(C), 1.5*U*_eq_(C19 and O1).

### Crystal data

**Table:**

C_19_H_16_N_2_O_3_	*F*_000_ = 672
*M**_r_* = 320.34	*D*_x_ = 1.368 Mg m^−^^3^
Monoclinic, *P*2_1_/*c*	Mo *K*α radiation λ = 0.71073 Å
Hall symbol: -P 2ybc	Cell parameters from 1378 reflections
*a* = 11.159 (2) Å	θ = 2.2–24.5º
*b* = 15.790 (3) Å	µ = 0.09 mm^−^^1^
*c* = 8.8300 (18) Å	*T* = 298 (2) K
β = 91.70 (3)º	Cut from a needle, colorless
*V* = 1555.2 (5) Å^3^	0.32 × 0.32 × 0.30 mm
*Z* = 4	

### Data collection

**Table:**

Bruker SMART CCD area-detector diffractometer	3550 independent reflections
Radiation source: fine-focus sealed tube	2161 reflections with *I* > 2σ(*I*)
Monochromator: graphite	*R*_int_ = 0.052
*T* = 298(2) K	θ_max_ = 27.5º
ω scans	θ_min_ = 1.8º
Absorption correction: multi-scan(SADABS; Sheldrick, 1996)	*h* = −14→14
*T*_min_ = 0.971, *T*_max_ = 0.972	*k* = −20→20
13232 measured reflections	*l* = −11→11

### Refinement

**Table:**

Refinement on *F*^2^	Secondary atom site location: difference Fourier map
Least-squares matrix: full	Hydrogen site location: inferred from neighbouring sites
*R*[*F*^2^ > 2σ(*F*^2^)] = 0.056	H atoms treated by a mixture of independent and constrained refinement
*wR*(*F*^2^) = 0.136	*w* = 1/[σ^2^(*F*_o_^2^) + (0.0391*P*)^2^ + 0.168*P*] where *P* = (*F*_o_^2^ + 2*F*_c_^2^)/3
*S* = 1.04	(Δ/σ)_max_ = 0.001
3550 reflections	Δρ_max_ = 0.18 e Å^−^^3^
222 parameters	Δρ_min_ = −0.20 e Å^−^^3^
1 restraint	Extinction correction: none
Primary atom site location: structure-invariant direct methods	

### Special details

**Table:**

Geometry. All esds (except the esd in the dihedral angle between two l.s. planes) are estimated using the full covariance matrix. The cell esds are taken into account individually in the estimation of esds in distances, angles and torsion angles; correlations between esds in cell parameters are only used when they are defined by crystal symmetry. An approximate (isotropic) treatment of cell esds is used for estimating esds involving l.s. planes.
Refinement. Refinement of F^2^ against ALL reflections. The weighted R-factor wR and goodness of fit S are based on F^2^, conventional R-factors R are based on F, with F set to zero for negative F^2^. The threshold expression of F^2^ > 2sigma(F^2^) is used only for calculating R-factors(gt) etc. and is not relevant to the choice of reflections for refinement. R-factors based on F^2^ are statistically about twice as large as those based on F, and R- factors based on ALL data will be even larger.

### Fractional atomic coordinates and isotropic or equivalent isotropic displacement parameters (Å^2^)

**Table:**

	*x*	*y*	*z*	*U*_iso_*/*U*_eq_	
O1	0.44682 (14)	0.12307 (9)	0.77419 (16)	0.0569 (4)	
H1	0.4089	0.1584	0.7244	0.085*	
O2	0.24817 (13)	0.32717 (8)	0.67361 (14)	0.0515 (4)	
O3	0.02184 (13)	0.57256 (9)	0.17402 (17)	0.0602 (5)	
N1	0.30518 (14)	0.17750 (9)	0.55897 (17)	0.0415 (4)	
N2	0.25139 (15)	0.24056 (10)	0.47098 (17)	0.0423 (4)	
C1	0.35505 (16)	0.03145 (12)	0.5862 (2)	0.0362 (4)	
C2	0.42732 (17)	0.04559 (13)	0.7145 (2)	0.0431 (5)	
C3	0.48691 (18)	−0.02246 (15)	0.7874 (2)	0.0520 (6)	
H3	0.5370	−0.0121	0.8715	0.062*	
C4	0.47221 (19)	−0.10234 (14)	0.7367 (2)	0.0526 (6)	
H4	0.5124	−0.1462	0.7868	0.063*	
C5	0.39740 (17)	−0.12109 (13)	0.6096 (2)	0.0434 (5)	
C6	0.3824 (2)	−0.20495 (13)	0.5558 (3)	0.0561 (6)	
H6	0.4230	−0.2489	0.6051	0.067*	
C7	0.3101 (2)	−0.22238 (14)	0.4342 (3)	0.0608 (6)	
H7	0.3007	−0.2779	0.4007	0.073*	
C8	0.2498 (2)	−0.15638 (14)	0.3591 (3)	0.0586 (6)	
H8	0.2005	−0.1683	0.2750	0.070*	
C9	0.26197 (18)	−0.07449 (12)	0.4075 (2)	0.0458 (5)	
H9	0.2198	−0.0318	0.3566	0.055*	
C10	0.33734 (16)	−0.05348 (11)	0.5331 (2)	0.0371 (5)	
C11	0.29956 (17)	0.10221 (12)	0.5053 (2)	0.0388 (5)	
H11	0.2594	0.0926	0.4131	0.047*	
C12	0.22727 (17)	0.31534 (11)	0.5375 (2)	0.0376 (5)	
C13	0.17287 (17)	0.38188 (11)	0.4404 (2)	0.0365 (4)	
C14	0.18459 (18)	0.46552 (12)	0.4857 (2)	0.0454 (5)	
H14	0.2259	0.4778	0.5761	0.054*	
C15	0.13651 (19)	0.53130 (12)	0.4000 (2)	0.0493 (5)	
H15	0.1466	0.5871	0.4317	0.059*	
C16	0.07353 (17)	0.51333 (12)	0.2674 (2)	0.0427 (5)	
C17	0.05869 (18)	0.43022 (12)	0.2214 (2)	0.0453 (5)	
H17	0.0147	0.4180	0.1329	0.054*	
C18	0.10864 (17)	0.36569 (12)	0.3058 (2)	0.0406 (5)	
H18	0.0995	0.3101	0.2725	0.049*	
C19	0.0515 (2)	0.65852 (13)	0.2009 (3)	0.0685 (7)	
H19A	0.0224	0.6755	0.2974	0.103*	
H19B	0.0153	0.6931	0.1227	0.103*	
H19C	0.1370	0.6652	0.2007	0.103*	
H2	0.251 (2)	0.2301 (14)	0.3703 (12)	0.080*	

### Atomic displacement parameters (Å^2^)

**Table:**

	*U*^11^	*U*^22^	*U*^33^	*U*^12^	*U*^13^	*U*^23^
O1	0.0614 (11)	0.0580 (10)	0.0505 (10)	−0.0051 (8)	−0.0100 (8)	−0.0063 (8)
O2	0.0791 (11)	0.0422 (8)	0.0326 (8)	0.0005 (7)	−0.0063 (7)	−0.0019 (6)
O3	0.0683 (11)	0.0440 (9)	0.0674 (11)	0.0126 (7)	−0.0113 (8)	0.0084 (8)
N1	0.0508 (11)	0.0356 (9)	0.0380 (9)	0.0008 (8)	0.0009 (8)	0.0047 (7)
N2	0.0608 (11)	0.0320 (9)	0.0339 (9)	0.0046 (8)	−0.0029 (9)	0.0013 (7)
C1	0.0350 (10)	0.0388 (11)	0.0350 (11)	0.0019 (8)	0.0038 (8)	0.0062 (8)
C2	0.0391 (11)	0.0500 (13)	0.0405 (12)	−0.0026 (10)	0.0036 (9)	0.0034 (10)
C3	0.0414 (12)	0.0728 (16)	0.0413 (13)	0.0038 (11)	−0.0065 (10)	0.0114 (11)
C4	0.0465 (13)	0.0549 (14)	0.0564 (14)	0.0122 (10)	0.0035 (11)	0.0204 (11)
C5	0.0371 (11)	0.0461 (12)	0.0474 (12)	0.0058 (9)	0.0077 (9)	0.0119 (10)
C6	0.0589 (15)	0.0399 (13)	0.0700 (16)	0.0138 (11)	0.0121 (12)	0.0144 (11)
C7	0.0684 (16)	0.0394 (13)	0.0750 (17)	0.0035 (11)	0.0085 (14)	−0.0022 (12)
C8	0.0658 (16)	0.0512 (14)	0.0586 (15)	−0.0033 (11)	−0.0020 (12)	−0.0065 (11)
C9	0.0500 (13)	0.0380 (11)	0.0492 (13)	0.0013 (9)	−0.0004 (10)	0.0014 (9)
C10	0.0349 (11)	0.0396 (11)	0.0370 (11)	0.0022 (9)	0.0062 (9)	0.0069 (9)
C11	0.0421 (12)	0.0395 (11)	0.0348 (11)	−0.0008 (9)	0.0006 (9)	0.0035 (9)
C12	0.0423 (11)	0.0359 (11)	0.0346 (11)	−0.0056 (8)	0.0005 (9)	−0.0003 (8)
C13	0.0397 (11)	0.0349 (10)	0.0350 (11)	−0.0017 (8)	0.0033 (8)	−0.0002 (8)
C14	0.0545 (13)	0.0409 (12)	0.0403 (12)	−0.0017 (10)	−0.0045 (10)	−0.0061 (9)
C15	0.0609 (14)	0.0322 (11)	0.0547 (14)	0.0017 (10)	−0.0011 (11)	−0.0036 (10)
C16	0.0408 (11)	0.0412 (12)	0.0458 (12)	0.0050 (9)	−0.0008 (9)	0.0032 (9)
C17	0.0454 (13)	0.0479 (13)	0.0421 (12)	−0.0002 (10)	−0.0077 (10)	−0.0004 (10)
C18	0.0467 (12)	0.0351 (11)	0.0397 (11)	−0.0029 (9)	−0.0020 (9)	−0.0046 (9)
C19	0.0857 (19)	0.0414 (13)	0.0784 (18)	0.0172 (12)	0.0019 (14)	0.0076 (12)

### Geometric parameters (Å, °)

**Table:**

O1—C2	1.347 (2)	C7—C8	1.397 (3)
O1—H1	0.8200	C7—H7	0.9300
O2—C12	1.231 (2)	C8—C9	1.367 (3)
O3—C16	1.363 (2)	C8—H8	0.9300
O3—C19	1.415 (2)	C9—C10	1.411 (3)
N1—C11	1.281 (2)	C9—H9	0.9300
N1—N2	1.388 (2)	C11—H11	0.9300
N2—C12	1.350 (2)	C12—C13	1.475 (3)
N2—H2	0.904 (9)	C13—C14	1.385 (2)
C1—C2	1.389 (3)	C13—C18	1.394 (3)
C1—C10	1.432 (2)	C14—C15	1.384 (3)
C1—C11	1.455 (2)	C14—H14	0.9300
C2—C3	1.409 (3)	C15—C16	1.377 (3)
C3—C4	1.347 (3)	C15—H15	0.9300
C3—H3	0.9300	C16—C17	1.382 (3)
C4—C5	1.410 (3)	C17—C18	1.371 (3)
C4—H4	0.9300	C17—H17	0.9300
C5—C6	1.415 (3)	C18—H18	0.9300
C5—C10	1.421 (3)	C19—H19A	0.9600
C6—C7	1.352 (3)	C19—H19B	0.9600
C6—H6	0.9300	C19—H19C	0.9600
			
C2—O1—H1	109.5	C9—C10—C5	117.31 (18)
C16—O3—C19	117.63 (17)	C9—C10—C1	123.40 (17)
C11—N1—N2	116.28 (16)	C5—C10—C1	119.29 (18)
C12—N2—N1	118.18 (15)	N1—C11—C1	121.04 (18)
C12—N2—H2	126.4 (15)	N1—C11—H11	119.5
N1—N2—H2	114.0 (15)	C1—C11—H11	119.5
C2—C1—C10	119.24 (17)	O2—C12—N2	121.54 (17)
C2—C1—C11	120.36 (18)	O2—C12—C13	121.46 (17)
C10—C1—C11	120.39 (17)	N2—C12—C13	116.99 (16)
O1—C2—C1	123.23 (18)	C14—C13—C18	117.57 (18)
O1—C2—C3	116.45 (18)	C14—C13—C12	118.53 (17)
C1—C2—C3	120.31 (19)	C18—C13—C12	123.89 (17)
C4—C3—C2	120.7 (2)	C15—C14—C13	121.75 (19)
C4—C3—H3	119.6	C15—C14—H14	119.1
C2—C3—H3	119.6	C13—C14—H14	119.1
C3—C4—C5	121.63 (19)	C16—C15—C14	119.34 (19)
C3—C4—H4	119.2	C16—C15—H15	120.3
C5—C4—H4	119.2	C14—C15—H15	120.3
C4—C5—C6	121.66 (19)	O3—C16—C15	124.67 (18)
C4—C5—C10	118.73 (19)	O3—C16—C17	115.39 (18)
C6—C5—C10	119.6 (2)	C15—C16—C17	119.94 (18)
C7—C6—C5	121.3 (2)	C18—C17—C16	120.17 (18)
C7—C6—H6	119.4	C18—C17—H17	119.9
C5—C6—H6	119.4	C16—C17—H17	119.9
C6—C7—C8	119.5 (2)	C17—C18—C13	121.20 (18)
C6—C7—H7	120.2	C17—C18—H18	119.4
C8—C7—H7	120.2	C13—C18—H18	119.4
C9—C8—C7	121.0 (2)	O3—C19—H19A	109.5
C9—C8—H8	119.5	O3—C19—H19B	109.5
C7—C8—H8	119.5	H19A—C19—H19B	109.5
C8—C9—C10	121.29 (19)	O3—C19—H19C	109.5
C8—C9—H9	119.4	H19A—C19—H19C	109.5
C10—C9—H9	119.4	H19B—C19—H19C	109.5

### Hydrogen-bond geometry (Å, °)

**Table:**

*D*—H···*A*	*D*—H	H···*A*	*D*···*A*	*D*—H···*A*
O1—H1···N1	0.82	1.86	2.582 (2)	146
N2—H2···O2^i^	0.904 (9)	1.957 (12)	2.834 (2)	163 (2)

Symmetry codes: (i) *x*, −*y*+1/2, *z*−1/2.
